# Small-Angle Neutron Scattering Study of a Phosphatidylcholine–Phosphatidylethanolamine Mixture

**DOI:** 10.1021/acsomega.3c04164

**Published:** 2023-09-02

**Authors:** William T. Heller

**Affiliations:** Neutron Scattering Division, Oak Ridge National Laboratory, Oak Ridge, Tennessee 37831, United States

## Abstract

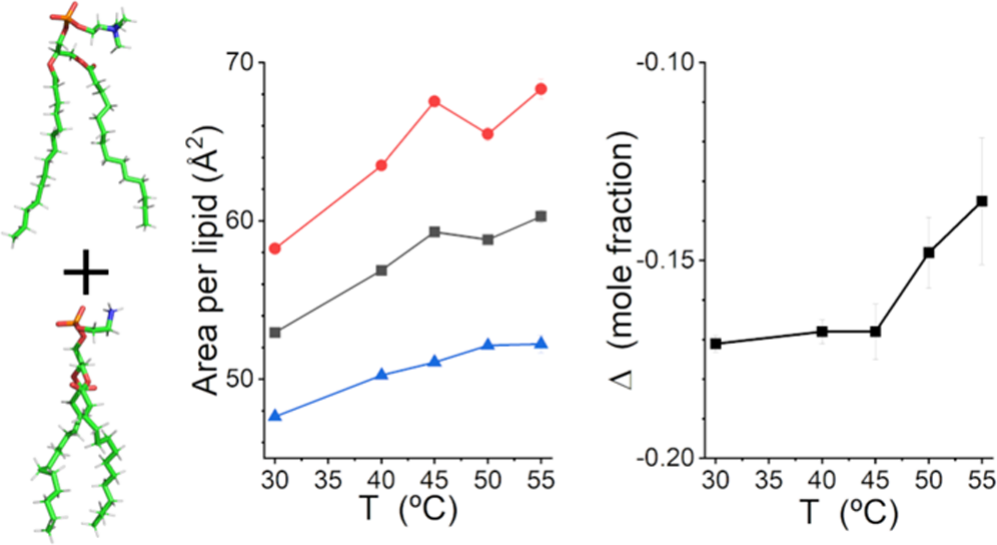

The properties of single-component phospholipid lipid bilayers have been extensively characterized. Natural cell membranes are not so simple, consisting of a diverse mixture of lipids and proteins. While having detailed structural information on complex membranes would be useful for understanding their structure and function, experimentally characterizing such membranes at a level of detail applied to model phospholipid bilayers is challenging. Here, small-angle neutron scattering with selective deuteration was used to characterize a binary lipid mixture composed of 1,2-dimyristoyl-3-*sn*-glycero-phosphatidylcholine and 1,2-dimyristoyl-3-*sn*-glycero-phosphatidylethanolamine. The data analysis provided the area per lipid in each leaflet as well as the asymmetry of the composition of the inner and outer leaflets of the bilayer. The results provide new insight into the structure of the lipid bilayer when this lipid mixture is used to prepare vesicles.

## 1 Introduction

The function of biological membranes arises from the diversity of proteins with specific functions and the many different lipids of which they are composed. For example, phosphatidylcholine and phosphatidylethanolamine lipids make up a large fraction of eukaryotic membranes, which also contain other phospholipids, cholesterol, sphingomyelin, and spingolipids.^1^ Phosphatidylglycerol lipids tend to be more prevalent in bacterial membranes than phosphatidylcholines.^2,3^ These compositionally complex membranes form well-structured bilayers as a result of the amphipathic nature of lipids. Hydrophobic effects are not the only governing factor driving the structure. Cells actively maintain different compositions for the inner and outer leaflets of the bilayer.^4,5^ Similarly, biological membranes are structured in a lateral sense.^6^ As a result, it is important to understand the structure of compositionally complex membranes.

Performing a detailed experimental study of lipid bilayers composed of more than one lipid species necessitates differentiating among the various lipids in the intact structure. Neutron scattering methods can do so through the use of selective deuterium labeling and contrast variation methods.^7^ Hydrogen (H) and its isotope deuterium (D) have very different neutron scattering lengths,^8^ even though they are indistinguishable when using X-rays. When combined with a neutron scattering technique that can probe length scales relevant to a lipid bilayer (sub-Å to μm), such as diffraction, small-angle neutron scattering (SANS), or neutron reflectometry (NR), it is possible to differentiate between lipids when more than one kind is present in the structure.

A study of the structure of lipid bilayer vesicles composed of a 7:3 molar mixture of deuterium-labeled 1,2-dimyristoyl-3-*sn*-glycero-phosphatidylcholine (DMPC) and unlabeled 1,2-dimyristoyl-3-*sn*-glycero-phosphatidylethanolamine (DMPE) is presented here. While the structures of DMPC lipid bilayers have been very extensively studied,^9−13^ the structures of DMPE lipid bilayers have not,^14−16^ primarily due to the strong intrinsic negative curvature of the lipid that results in it adopting the inverted hexagonal phase.^17−19^ In the present work, SANS was used to experimentally characterize the structure of unilamellar vesicles as a function of temperature. Deuterium labeling made it possible to determine if DMPE preferentially locates in the inner or outer leaflet of the bilayer of the vesicle, and it does so without introducing a spectroscopic label to the headgroup of either lipid used, which may alter the behavior of the lipid. The results shine improved light on the structure of unilamellar vesicles of this lipid mixture containing very different intrinsic curvatures by revealing a strong asymmetry in the composition of the inner and outer leaflets of the vesicle.

## 2 Experimental Section

### 2.1 Materials

Chain-perdeuterated DMPC (d54-DMPC) and DMPE were obtained from Avanti Polar Lipids (Alabaster, Alabama) and used without further purification. The d54-DMPC was supplied in CHCl_3_, while the DMPE was provided as a powder. A DMPE stock solution was prepared from the powder in a 65:35:8 CHCl_3_/methanol/water mixture. D_2_O was obtained from Cambridge Isotope Laboratories (Andover, Massachusetts). It was also used without further purification. Deionized water (18.2 MΩ) was used for H_2_O rather than the use of deuterium-depleted water. Deuterium is 0.2% of the natural abundance of hydrogen, so the choice is reasonable.

### 2.2 Vesicle Preparation

Vesicle preparation followed procedures used in previous studies.^20−24^ The lipids were mixed by liquid volume in glass vials to provide the desired molar ratio and total mass. Then, the solvent was blown off under a stream of dry N_2_. To remove any residual solvent, the lipid films in the vials were placed in a freeze drier for 16 h. Then, the samples were rehydrated in a 90% H_2_O/10% D_2_O mixture and vortexed until all lipids were suspended in solution. Next, the samples were subjected to freeze–thaw cycles using a −80 °C freezer and an oven set to 50 °C, which is near the gel-phase transition temperature of pure DMPE (51 °C)^25^ and above the expected transition temperature for this mixture.^26,27^ The third thaw cycle was followed by extrusion using a Mini-Extruder from Avanti Polar Lipids (Alabaster, Alabama). The polycarbonate filters used for extrusion had 100 nm pores. With the body of the extruder maintained at 50 °C on a hot plate, the solutions were passed through the filter at least 21 times. Care should be taken when extruding vesicles on a hot plate set to 50 °C because burns to the skin are possible.

### 2.3 Small-Angle Neutron Scattering Experiments

SANS data were collected using the EQ-SANS instrument, which is at the Spallation Neutron Source at Oak Ridge National Laboratory.^28^ Data were collected using a single instrument configuration with a sample-to-detector distance of 4 m and a neutron wavelength band of 2.5 to 6.1 Å. The sample temperature was maintained to within ±1 °C of the desired temperatures of 55, 50, 45, 40, and 30 °C, in that order, using a water bath. Each data set required 30 min to measure. The temperature was allowed to equilibrate for 60 min after it was changed to a new setting. Data reduction followed standard procedures implemented in the drtsans software package.^29^ The sample and solvent scattering data were corrected for empty cell, dark current signal, detector pixel response, wavelength-dependent incident beam flux, and sample transmission. During the data reduction process, a multiplicative scaling factor derived from a porous silica standard was applied, which resulted in the reduced data being output in absolute units of 1/cm.^30^ The reduced data were azimuthally averaged into the 1D SANS intensity profiles, *I*(*q*) vs *q* (*q* = 4πsin(θ)/λ). Finally, the 1D scattering data of the sample was corrected by subtracting the signal from the solvent.

### 2.4 Small-Angle Neutron Scattering Data Analysis

SANS data were fit using the 4-shell core–shell model that has been employed previously by this group for modeling lipid bilayer mixtures containing one species of deuterium-labeled lipid.^20,31^ However, the model was modified to be a self-consistent slab model^32−35^ that employed the sample composition and known physical parameters for the lipids. The model is described in detail below.

**Figure 1:**
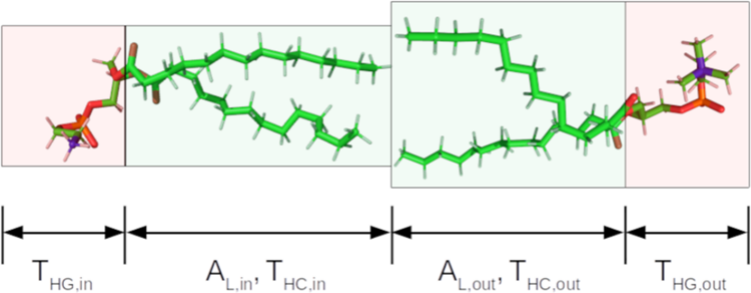
Schematic of the four-layer self-consistent slab model used for the SANS data analysis.

The four layers of the model are the inner headgroup (HG), the inner hydrocarbon core (HC), the outer HC, and the outer HG. A schematic of the structure is shown in Figure 1. The model is parametrized in terms of the areas per lipid of the inner and outer leaflets, *A*_L,in_ and *A*_L,out_, and Δ, the deviation of the DMPE content of the outer leaflet from the molar fraction of DMPE in the sample, which is denoted *f*. Negative values of Δ mean that the inner leaflet has more DMPE. Δ can adopt values from – *f* to *f*.

The thicknesses of the layers of the hydrocarbon core of the bilayer, *T*_HC,in_ and *T*_HC,out_, are calculated according to eqs 1 and 2, respectively.
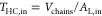
1
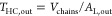
2*V*_chains_ is the volume of the dimyristoyl chains and is 780 Å^3^.^36^ For the thicknesses of the two headgroup regions of the bilayer, *T*_HG,in_ and *T*_HG,out_, a fixed value of 10 Å^37^ was employed to minimize the number of degrees of freedom used in the data analysis. The total bilayer thickness is given by *T*_tot_ = *T*_HG,in_ + *T*_HC,in_ + *T*_HC,out_ + *T*_HG,out_. The headgroup regions of the bilayer may contain water, the number of which can be determined using eqs 3 and 4.
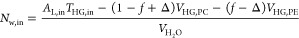
3
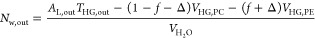
4The headgroup volumes are taken from previously published studies, specifically *V*_HG,PC_ = 320 Å^3^^36^ and *V*_HG,PE_ = 252 Å^3^.^38^ The volume of a water molecule, *V*_H_2_O_, is 30 Å^3^.

The scattering length densities, ρ, of the four layers in the model are calculated from the compositions of the layers and the structural parameters of the model. The scattering length densities of the inner and outer hydrocarbon regions, ρ_HC,in_ and ρ_HC,out_, are shown in eqs 5 and 6, respectively.
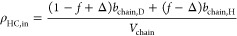
5
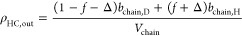
6The values of *b*_chain,D_ and *b*_chain,H_ are calculated from the scattering lengths of the isotopes^8^ and the chemical formulas. The scattering length densities of the inner and outer headgroup regions of the bilayer, ρ_HG,in_ and ρ_HG,out_, are calculated using eqs 7 and 8, respectively.
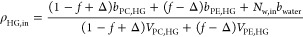
7
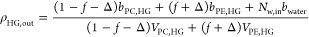
8Here, *b*_water_ = 0.9*b*_D_2_O_ + 0.1*b*_H_2_O_. The chemical formulas of the lipid headgroups were used to calculate their total scattering lengths, *b*_PC,HG_ and *b*_PE,HG_.

Data analysis also employed two additional models in order to test whether the bilayer was indeed asymmetric. The model remains the same as above, but constraints were applied to test different symmetric structures. The first symmetric structure tested fixed the value of Δ to be 0.0, meaning that the two leaflets of the bilayer had the same composition, i.e., each leaflet had 70 mol % d54-DMPC and 30 mol % DMPE. The *A*_L_ of the inner and outer leaflets were allowed to be different. In the second symmetric model tested, not only was Δ fixed to be 0.0, but the *A*_L_ of the two leaflets of the bilayer were also constrained to have the same value.

The model described above was implemented in the Python programming language. The process of fitting the model to the SANS data was accomplished using the sas-temper software,^39^ which leverages the sasmodels package of Sasview.^40^ Model intensity profiles were convoluted with the instrument resolution function of the EQ-SANS,^28^ which is a function of the sizes of the beam-defining apertures, the detector pixel sizes, and the wavelength distribution and uncertainty, before comparison against the data. This convolution smears out features in the calculated profiles, making them accurately represent what the instrument would measure. After an initial run of the fitting that produced 25 independent models was performed at each temperature, three additional refinement runs of the fitting were performed that employed narrowed constraints on the parameters. Each of these runs also produced 25 independent models. The averages and standard deviations of the free parameters used in the modeling found in the final run performed are presented here as the results. Most parameters began with physically reasonable but relaxed constraints during the first fitting. For example, *A*_L,in_ and *A*_L,out_ began by being constrained to be between 30 and 70 Å^2^. Tests of the fitting without a constraint on Δ, when it was allowed to vary, suggested that it had a preference for negative values, and so it was constrained to be so. This constraint was deemed to be reasonable because the intrinsic curvature of DMPE is considerably more negative than that of DMPC,^17−19^ in spite of a previous study of DMPC–DMPE mixtures.^27^

## 3 Results

**Figure 2:**
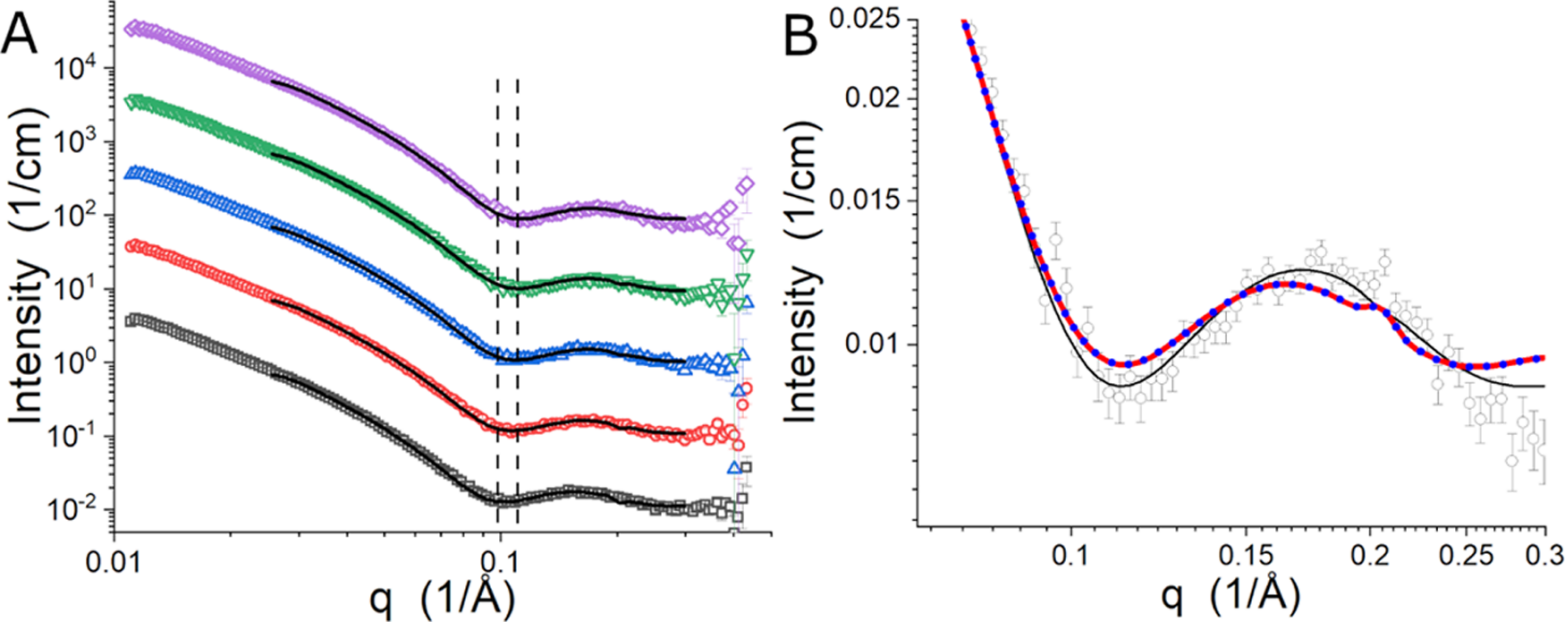
(A) SANS data collected for the 7:3 DMPC/DMPE vesicles as a function of temperature. The curves are 30 °C (black square), 40 °C (red circle), 45 °C (blue triangle), 50 °C (green down-pointing triangle), and 55 °C (magenta diamond). The SANS data set collected at 55 °C was presented previously in Figure 3A of reference 35 and is reused with permission Copyright 2022, by the author under the CC-BY 4.0 license (https://creativecommons.org/licenses/by/4.0/). The solid black lines in panel (A) are example model intensity profiles found during SANS data analysis. The data were offset from the 30 °C data for clarity. The vertical dashed lines are provided as guides for the eye. (B) Zoomed-in view of the SANS data collected at 55 °C (deg) and example best fit model curves for the three models described in Section 2.4. Three model curves are presented: the asymmetric model (solid black line), the symmetric composition model that can have different *A*_L_ values in the leaflets (solid red line), and the fully symmetric model (blue dotted line). The small, sharp feature in the two symmetric model curves near 0.20 Å^–1^ visible on the broad peak due to the bilayer structure is an artifact of the underlying mathematical functions of Python used in the model calculation that has been convoluted with the EQ-SANS *q*-resolution^28^ (see Section 2.4). Similar features at different locations in *q* can be seen in panel (A).

The SANS data and example fits to the data are shown in Figure 2A. The position of the minima that is near 0.1 Å^–1^ moves to higher *q* as the temperature increases. It shifts from *q* ∼ 0.099 Å^–1^ to *q* ∼ 0.111 Å^–1^. The change indicates that the bilayer thins with increasing temperature. The trend in the SANS data is consistent with the previously reported phase behavior of this lipid mixture over this temperature range.^25−27^

The asymmetric model fit curve for the 55 °C data set is shown in Figure 2B, with example fit curves found using the two different symmetric models described in the Section 2 to support the choice of the asymmetric model for fitting the data. Figure 2B focuses on the region of the data containing the minimum and oscillation, similar to the approach recently used by Frewein and co-workers when discussing the suitability of the model used for their data analysis.^41^ The fit curves for the two symmetric models employed are nearly indistinguishable but are quite different from the asymmetric model curve. While all three curves reproduce the position of the minimum (*q* ∼ 0.111 Å^–1^), the position of the subsequent local maximum (*q* ∼ 0.172 Å^–1^) in the SANS data is not fit well by the symmetric model curves, which have their local maximum at a lower *q*-value (*q* ∼ 0.164 Å^–1^) than the SANS data or the asymmetric model fit curve do. The width of this feature in the symmetric model curves is also too narrow to fit the feature in the SANS data. The symmetric models found by fitting the SANS data collected at the other measured temperatures (not shown) also fit the SANS data less well than the asymmetric model. Therefore, it is reasonable to conclude that using the asymmetric model described in the Section 2 is justified.

**Figure 3:**
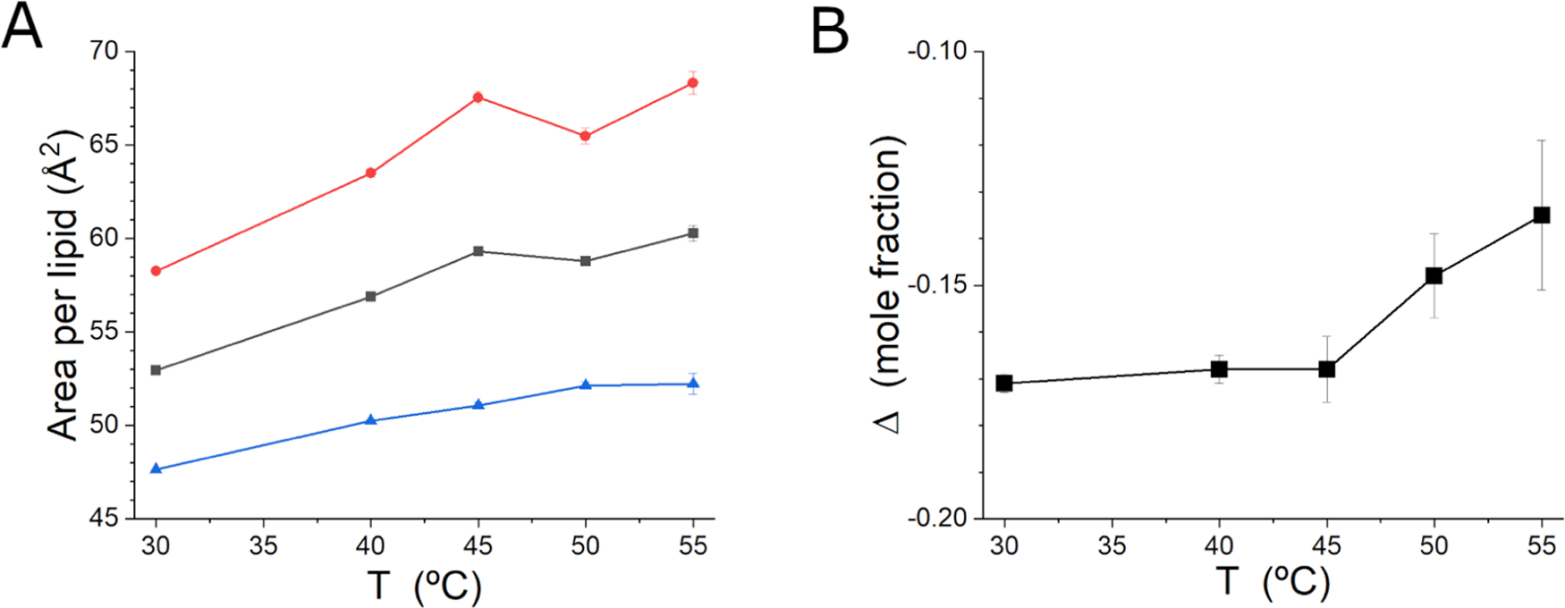
(A) *A*_L_ determined by fitting the SANS data and (B) Δ parameter as a function of temperature from the SANS data analysis. In panel (A), *A*_L,in_ and *A*_L,out_ from the SANS data fitting are in red and blue, respectively. The average *A*_L,ave_ is black.

The model SANS profiles reproduce the features in the SANS data well (Figure 2). The fitting results are presented in Table 1, which include both free parameters used for the fitting and relevant derived parameters. The *A*_L_ values determined from the data analysis are presented in Figure 3A. As can be seen in Figure 3A and Table 1, *A*_L,in_ is always larger than *A*_L,out_. There is a relatively large difference in *A*_L,in_ and *A*_L,out_, being ∼15 Å^2^ at the highest temperatures studied. *A*_L,in_ also increases more rapidly with temperature than *A*_L,out_, even though it levels off at the highest temperatures studied. The trend in the latter is monotonic, while the trend in the former is not, which may be evidence that the “liquid + solid” phase is present in that particular leaflet at the lowest temperature. Both bulk^26^ and unilamellar vesicle^27^ phase diagrams show that a 7:3 mixture of DMPC and DMPE are in the “liquid + solid” portion of the phase diagram at the lowest temperature studied here, while the rest of the temperatures studied are in the L_α_ phase.

**Table 1:** Table of Parameters from SANS Fitting^a^

parameter name	30 °C	40 °C	45 °C	50 °C	55 °C
*A*_L,in_ (Å)^2^	58.3(2)	63.5(2)	67.5(3)	65.5(4)	68.3(6)
*A*_L,out_ (Å)^2^	47.6(1)	50.3(1)	51.1(2)	52.1(2)	52.2(6)
*A*_L,ave_*(Å)^2^	52.9(1)	56.8(1)	59.3(2)	58.8(2)	60.3(6)
*T*_HC,in_*(Å)	13.4(1)	12.3(1)	11.5(1)	11.9(2)	11.4(2)
*T*_HC,out_*(Å)	16.4(1)	15.5(1)	15.3(1)	15.0(1)	14.9(3)
*T*_tot_*(Å)	49.8(1)	47.8(1)	46.8(2)	46.9(2)	46.3(4)
*N*_w,in_*(Å)	9.8(1)	11.6(1)	12.9(1)	12.2(2)	13.1(2)
*N*_w,out_*(Å)	9.0(1)	10.8(1)	12.2(1)	11.5(1)	12.5(2)
Δ (mole frac.)	–0.171(2)	–0.168(3)	–0.167(7)	–0.148(9)	–0.135(16)

a A “*” denotes a parameter derived from the fitting results for the free parameters. The standard deviation in the value found by sas-temper^39^ is shown in parentheses and is for the last digit or digits.

The *T*_HC,in_ and *T*_HC,out_ values that were derived from the *A*_L_ obtained from the SANS fitting are presented in Table 1, as is *T*_tot_. The trends follow those in *A*_L_ for both leaflets because these parameters are derived from the *A*_L_. The two leaflets differ in thickness by 3–4 Å across the entire temperature range studied. The smallest difference is observed when the lipids are in the “liquid + solid” region of the phase diagram at 30 °C. The total thickness of the bilayer is 3.6 Å thinner at 55 °C than it is at 30 °C. The total number of water molecules found to be associated with the lipid headgroups, per the self-consistent slab model,^32−35^ described in Section 2.4, is also presented in the table.

**Figure 4:**
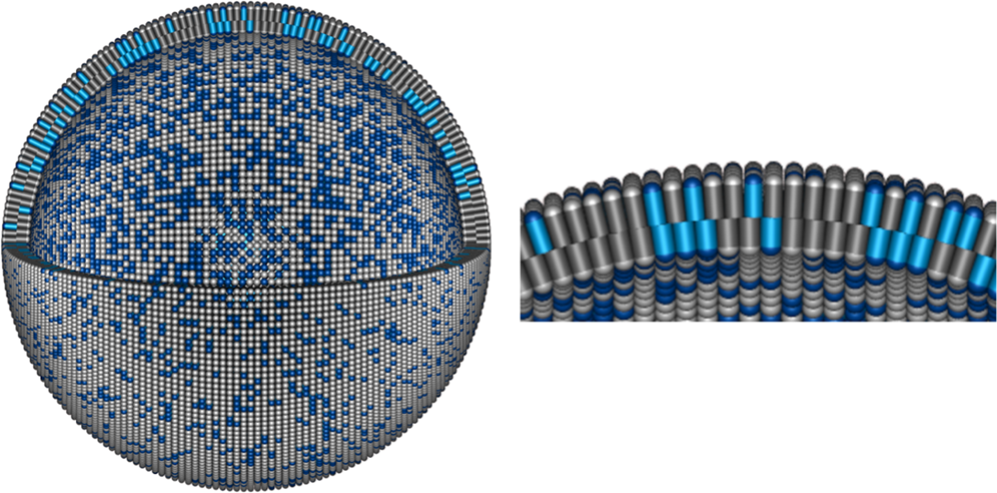
Simplified representation of the vesicle structure at 55 °C. DMPC is colored white and gray, while DMPE is colored shades of blue. The left image shows the vesicle at a scale with a 90° wedge removed to reveal the content of the inner and outer leaflets. The right image shows a zoomed-in view of the top of the vesicle that better presents the differences in *A*_L_ and leaflet thicknesses of the inner and outer leaflets. The image was rendered using Persistence of Vision Raytracer software^42^ from an input file produced by software written for the generation of this kind of vesicle schematic that has been used previously.^31,35^ The software was modified to show the differences in the structures of the inner and outer leaflets.

The relatively strong asymmetry in the content of the inner and outer leaflets of the bilayer can be seen in the plot of Δ as a function of the temperature in Figure 3B. *A*_L,in_ is the larger of the two lipid areas, even though Δ indicates that the inner leaflet has considerably more DMPE than the outer leaflet. Δ is considerably less negative at the two highest temperatures studied than it is at the lower temperatures. A single sample was used for the entire experiment, and the temperature series was measured from high to low temperature; therefore, the result implies that some lipids moved between leaflets of the bilayer as the temperature decreased. A simplified representation of the vesicle and the bilayer at 55 °C found by the SANS data analysis that illustrates the asymmetry in the structure is shown in Figure 4. The vesicle radius, bilayer thickness, and *A*_L_ are presented to scale. The zoomed-in view illustrates that the curvature of the bilayer is non-negligible, even on a relatively local scale.

The intrinsic curvatures, *c*_0_, of the inner and outer leaflets of the vesicle can be estimated from the values of the intrinsic curvatures of each lipid by using eq 2 of the review by Dymond^19^ and the mole fractions of each lipid. For DMPC at 35 °C, *c*_0_ = 0.007 ± 0.002 Å^–1^,^43^ while for DMPE, *c*_0_ = −0.0314 ± 0.0006 Å^–1^^44^ at 80 °C. Table 2 presents the values determined from Δ as a function of temperature using eq 2 of the review by Dymond.^19^ It is important to note that *c*_0_ generally decreases with increasing temperature above the phase transition.^19^ However, the rates of change of *c*_0_ with temperature of DMPC and DMPE were not presented in the recent review by Dymond.^19^ Applying the logic of the general trend would result in the *c*_0_ for DMPE being less negative than the value used for the calculations presented here and the value of DMPC being more negative at most of the temperatures studied here, although it is not ideal to extrapolate such behavior for a mixture of lipids. It is reasonable to conclude that the *c*_0_ values for the inner and outer leaflets would be closer if it were possible to correct for the temperature. As a result, the values shown in Table 2 should be considered estimates.

**Table 2:** Intrinsic Curvature of the Inner and Outer Leaflets Estimated from the Value of Δ Determined from the SANS Fitting^a^

temperature	*c*_0,in_ (Å^–1^)	*c*_0,out_ (Å^–1^)
30 °C	–0.0111	0.0020
40 °C	–0.0110	0.0019
45 °C	–0.0109	0.0019
50 °C	–0.0102	0.0012
55 °C	–0.0097	0.0007

a A negative value means that the headgroups face toward the center of the curvature, while a positive value means that they point away from it.

## 4 Discussion

The present results provide new information about the structure of the lipid bilayer vesicles made of a 7:3 molar mixture of DMPC and DMPE and how it depends on the temperature. The use of deuterium-labeled DMPC made it possible to observe differences in the structures of the two leaflets of the bialyer.^7,35^ The *A*_L_ of the lipids in the inner leaflet of the bilayer displays a stronger temperature dependence than that of the outer one. The SANS results also revealed considerable asymmetry in the composition of the inner and outer leaflets of the bilayer, which Figure 4 highlights clearly. The amount of asymmetry found here also depends on temperature. Δ becomes ∼4 mol % more positive with increasing temperature.

While the SANS data revealed that the compositions of the inner and outer leaflets of the bilayer were not identical, there was no indication that the samples were not laterally homogeneous at the length scales sampled by the SANS measurements, which ranged from distances comparable to the radius of the vesicle to those less than the thickness of the bilayer. Lateral structures in the bilayer have been observed by SANS.^45−49^ Such lateral structures can take the form of lipid rafts, being large-scale patches (≥50 Å in size) enriched in specific lipids,^45,48,49^ which must have existed as a persistent population over the course of the SANS measurement time to be observable in the data. These structures create a SANS signal that takes the form of a peaklike feature at low-*q*, which is not found in any of the SANS data presented herein. Evidence of lateral structuring can also be present in data at shorter length scales and may not result from raft-like domains. In vesicles made of DMPC and cholesterol, the presence of two different thicknesses in the vesicles was inferred from the SANS data.^46,47^ In both studies, the first oscillation in the SANS data (the equivalent of the feature highlighted in Figure 2B) was noticeably flattened and could not be modeled using a bilayer with a single thickness, suggesting lateral segregation. Again, this kind of structure must exist as a persistent population over time scales comparable to the measurement time to be observable. The SANS data of these earlier studies of DMPC and cholesterol did not possess any features at lower *q* that were indicative of larger-scale structures.^46,47^ The SANS data presented herein do not display a clear distortion of this feature that suggests that the vesicle is not uniform in a lateral sense. Based on these possibilities and the *q*-range sampled in the SANS measurements and fit during data analysis, the vesicles are laterally homogeneous from ∼20 Å to over ∼250 Å.

The average *A*_L_ for the 7:3 DMPC/DMPE mixture studied here by SANS ranges from 52.9 to 60.3 Å^2^ with increasing temperature, which is below the *A*_L_ for pure L_α_ phase DMPC. The *A*_L_ of gel-phase DMPC is 47.2 Å^2^,^10^ while fluid (*L*_α_) phase DMPC has an *A*_L_ of 59.9 Å^2^ at 30 °C, 63.3 Å^2^ at 50 °C, and 65.7 Å^2^ at 60 °C.^12^ Gel-phase DMPE has an experimentally determined *A*_L_ that ranges from ∼40.5^16,50^ to ∼56.1 Å^2^,^51^ while the *A*_L_ of fluid-phase DMPE was determined experimentally to be 63–70 Å^2^.^15^ The large ranges of the DMPE *A*_L_ is somewhat surprising, but in light of the well-characterized *A*_L_ of DMPC, the *A*_L_ of DMPE is more likely to be near the lower ends of the reported ranges instead of near the upper ends. The present results for the *A*_L_ of the two leaflets are reasonable and are in-line in terms of the overall magnitude of the differences from the pure lipids with other studies of mixtures of PC and PE lipids.^52−55^ In light of the known phase behavior of DMPC/DMPE mixtures,^26^ including for small unilamellar vesicles,^27^ the sample at 30 °C was in the “liquid + solid” coexistence region of the phase diagram. The higher temperatures studied were well within the liquid crystalline *L*_α_ phase.^26,27^ The average *A*_L_ in Table 1 supports this conclusion, but the *A*_L_ of each leaflet suggests that the outer leaflet is considerably more ordered than a typical liquid crystalline phase at all temperatures studied, while the inner leaflet is less ordered.

The differences in *A*_L_ for the two vesicle leaflets found by SANS make reasonable sense physically if one considers the vesicle radius and leaflet thicknesses. Consider the structure found at 55 °C (Table 1). The area of the spheres at the inner surface, at the position of the terminal methyl groups of the lipid acyl chains, and at the outer surface are 2.88 × 10^6^, 3.14 × 10^6^, and 3.46 × 10^6^ Å^2^, respectively. The inner and outer surface areas are 8.4% smaller and 10.2% larger than the area of the sphere at the center of the bilayer, respectively. The differences in *A*_L_ are physically reasonable when the differences in available surface area in the structure are taken into account. The inner leaflet’s lipid acyl chains must expand to make up for the difference between the area available at the inner surface and at the methyl groups, making it thinner. Similarly, those of the lipids in the outer leaflet must pack more tightly to ensure that water is excluded from the hydrocarbon core. As a result, the outer leaflet of the bilayer becomes thicker.

Previous experiments revealed asymmetry in DMPC/DMPE mixtures.^27^ While asymmetric bilayers were observed, the outer leaflet of the bilayer was found to be considerably enriched in DMPE (see Figure 3 of ref (27)), unlike the present study. The extent of enrichment of the outer leaflet was comparable to that of the inner leaflet seen in the present study. The experiments performed previously differed considerably from those in this study considerably. The vesicles were prepared by sonication followed by ultracentrifugation, which produced smaller vesicles than were studied here.^27^ The assay used to determine the PE content of the outer layer involves adding 0.2 mL of a 0.8 M NaHCO_3_ (pH 8.5) solution to the sample, followed by an additional 0.4 mL of a 1.2% Triton X-100 solution. Thus, the procedure used in the previous work is very different than that employed in the present study. Further, the PE headgroup adopts a slight negative charge at basic pH,^56^ and charge repulsion was one of the reasons suggested by the authors of the previous study for the asymmetry observed.^27^ The present study used D_2_O as is, with no additional salt or buffering (pH ∼ 6.7, i.e., pD ∼ 7.1), so the DMPE should be neutral in the present study. Interestingly, an earlier study of mixtures of PC and PE lipids derived from egg yolks, which contain more unsaturated acyl chains, found enrichment of PE in the inner leaflet of the bilayer^57^ using a very similar experimental procedure to the study of DMPC/DMPE mixtures.^27^ The different results were attributed to the egg yolk PE having a stronger negative radius of curvature than DMPE.^27^

## 5 Conclusions

The characterization of the multicomponent lipid bilayer vesicles presented here provides new insight into the structure of lipid bilayer vesicles that are binary mixtures of lipids having very different curvatures. Differences in leaflet composition were resolved. Importantly, differences in the inner and outer *A*_L_ could be resolved. The results make it very clear that SANS and contrast variation methods^7,35^ are powerful tools for performing detailed structural studies of mixed-composition lipid bilayer vesicles in order to capture differences in the composition of the bilayer leaflets and any asymmetry that may exist.

## Notes

**Notice of Copyright** This manuscript has been authored by UT-Battelle, LLC, under Contract DE-AC05-00OR22725 with the U.S. Department of Energy (DOE). The U.S. Government retains and the publisher, by accepting the article for publication, acknowledges that the U.S. Government retains a nonexclusive, paid-up, irrevocable, worldwide license to publish or reproduce the published form of this manuscript or allow others to do so for U.S. Government purposes. DOE will provide public access to these results of federally sponsored research in accordance with the DOE Public Access Plan (http://energy.gov/downloads/doe-public-access-plan).
